# The development of creative imagination and symbolic thinking through thematic social role-play activity

**DOI:** 10.3389/fpsyg.2026.1916508

**Published:** 2026-10-22

**Authors:** Claudia Ximena González-Moreno, Yulia Solovieva

**Affiliations:** 1 Pontificial Javeriana University, Bogota, Colombia; 2 Meritorious Autonomous University of Puebla, Puebla, Mexico; 3 Autonomous University of Tlaxcala, Tlaxcala, Mexico; 4 Federal Center of Psychological and Multidisciplinary Research, Moscow, Russia

**Keywords:** child development, creative imagination, personality development, symbolic thinking, thematic social role-play

## Abstract

Grounded in Vygotsky's cultural-historical paradigm, this article presents a theoretical analysis of the role of thematic social role-play in preschool psychological development, positioning it not merely as a recreational activity but as the leading activity driving psychological maturation. Within this framework, we examine how high-level play serves as the critical foundation for the genesis of creative imagination and symbolic thought—all essential components of the developing child's psychological neoformations. Specifically, we analyze the trajectory of adult-guided orientation, illustrating its necessity in transforming initial, fragmented play behaviors into advanced, mature role-play characterized by reflective initiative, internal rules, and symbolic verbal actions. Furthermore, we delineate the transition from object-substituted actions to deep psychological internalization. Based on this theoretical synthesis, we hypothesize that a child's readiness for formal school learning is a direct function of the semiotic and structural complexity of their thematic social role-play. We propose that the more intentional, collaborative, and creative the play behavior, the stronger the cognitive, volitional, and social foundations for subsequent academic success. Finally, we argue for an educational shift that prioritizes structured, high-level thematic social role-play not as a leisure choice, but as a core, non-negotiable developmental necessity in early childhood education.

## Introduction

“*Play is the source of development and creates the zone of proximal development”*

             *Vygotsky*

The development of creative imagination and symbolic thinking during preschool age has long-term effects on a child's personality, particularly on their socioemotional, creative, and self-regulatory development. Research has shown that this growth depends heavily on the quality of thematic social role-play, which is primarily determined by four factors: (1) the clarity of the objectives set by the adult, considering the children's psychological developmental needs; (2) the relevance of the thematic situations to the children; (3) the level of emotional involvement of the participants; and (4) the feedback provided by the adult. Within this play, adult intervention and guidance are designed to generate, mediate, and support the children's initiative, creativity, and choices (Zuckerman, 2007). Role-play serves as the pathway to the complex development of creative imagination and symbolic thinking. It is an activity where children adopt the social functions of adults and, within imaginary conditions recreated by themselves, model social relations and their underlying meanings (Smirnova, 2003). By stepping into a role, the child is compelled to follow its implicit rules, allowing them to voluntarily internalize moral norms, empathy, and self-regulation. Through this process, children assimilate both the content of human moral standards and the dynamics of interpersonal relationships (Elkonin, 1980).

This activity is based on the theoretical premise that social relationships act as psychological tools that formalize thought into thematic units, which develop through joint interactions between adults and children. Creating a shared imaginative environment in play begins with an engaging social situation that fosters symbolic actions, creative problem-solving, and perspective-taking. Adult involvement is crucial in guiding children to develop both cooperative play skills and the capacity to play independently (Hagen and Heldal, 2025). Furthermore, participants build on each other's ideas, ensuring the activity remains meaningful and open to multiple possibilities (Pramling et al., 2025).

The potential of social role-play to enhance the development of creative imagination and symbolic thinking is implicit in several research studies (Elkonin, 1980; González-Moreno and Solovieva, 2025). These psychological formations during the preschool years lay the foundation for future academic success and lifelong learning. For instance, when a child uses a wooden block as a telephone or an airplane, they break the literal link to the physical object. The child no longer reacts to the object based on its objective properties, but rather on what it represents in their mind.

Likewise, children who have developed creative imagination and symbolic thinking during preschool can, upon reaching school age, invent stories, develop narratives according to a thematic chart, or draw imaginary narrative situations. This possesses high prognostic value for later stages of life, including for children with special educational needs. For example, research has shown that play allows children with autism to develop creative imagination and symbolic function (González-Moreno, 2015, 2023), which subsequently contributes to learning to read and write at school age.

Thematic social role-play supports the child's psychological transition from preschool to school-age learning (González-Moreno and Solovieva, 2024, 2025). Therefore, a crucial task for the teacher is to understand the relational dynamics between these two stages of development (preschool and school), as well as the needs of each and the causal relationship between them. For example, before starting school, children require a certain level of mastery of symbolic means and the ability to consider the perspective of others (Elkonin, 1989). Likewise, a specific level of communication is required for children to submit to rules, orient themselves toward social norms, live together, and develop the capacity for mutual understanding (Lisina, 1986).

The primary goal of preschool education is to ensure an optimal level of psychological readiness for the transition to formal schooling. This involves developing key psychological milestones that emerge at the end of the preschool period. Specifically, the objectives focus on fostering these new psychological formations through the development of:

- Voluntary behavior and self-regulation: cultivating respect for rules and norms, alongside the ability to reflect when setting personal goals across various activities.
- Reflective communication: encouraging a thoughtful attitude toward interactions with both peers and adults, fostering broad and positive social relationships.
- Imagination and empathy: enhancing the ability to anticipate social behaviors (one's own and others') while producing and sharing imaginary situations in playful, creative settings.
- Socioemotional and personality growth: nurturing motivation for social exchange through compassion, empathy, respect, understanding, and mutual support in daily life.
- Cognitive motivation: stimulating a broad interest in knowledge and future systematic learning, manifested through curiosity and a proactive attitude toward information in society.

Play serves as the primary mechanism in early childhood for rehearsing and modeling real-life scenarios (Bodrova et al., 2013). Through play-based activities, children develop impulse control, practice empathy, and learn to manage frustration within a safe, adaptable environment. Shared and guided play act as an essential bridge to independent play. Furthermore, collaborative play modeled by adults provides the structure necessary for children to identify, label and regulate their own emotional states.

Play also fosters so-called 21st-century skills: collaboration, communication, critical thinking, confidence, and creativity (Fletcher et al., 2024). For an activity to be considered true play, it must meet the following criteria (Hirsh-Pasek et al., 2009):

- Active engagement: the child engages with enthusiasm, remaining mentally active and deeply involved in the activity.
- Meaningful experiences: the activity connects new experiences to the child's existing knowledge and world.
- Social interaction: the play allows for meaningful connections and communication with peers or adults.
- Iterative thinking: the activity encourages experimentation, trial and error and problem-solving.
- Joyful engagement: the child finds the process intrinsically motivating, satisfying and fun.

The preschool years introduce essential qualitative changes—such as symbolic function and self-regulation—that lay the groundwork for school-based learning. Adequate preparation ensures children possess the cognitive tools required to transition from practical actions to more abstract mental operations. School readiness aims to foster a conscious, reflective approach to one's own actions. This enables students to plan and control their behavior instead of acting on impulse, promoting harmonious coexistence and the achievement of school goals.

## Importance of thematic social role-play

Children's play is based on imagination, culture, and the active participation of adults with children (Lindqvist, 1996). This preschool activity aims to help children develop autonomy in play, meaning they become able to create role-play, follow the rules of the play, learn to take turns in conversation, and solve problems that arise during play.

Thematic social role-play is an activity that unfold over time and enables interaction between children through the inclusion of a common goal, which is achieved thanks to the union of mutual efforts from all participants (Elkonin, 1999). Role-play is the leading activity in preschool age (Elkonin, 1980) and possesses an interpsychological character (Vygotsky, 1995). This means that this activity is shared and enriched through dialogue, the negotiation of rules, empathy in assuming roles, and social and communicative interaction. Play is based on an active communicative exchange between participants. Together, children and adults create an imaginary situation, negotiate rules, and resolve conflicts, allowing them to move from group interaction to the individual internalization of social norms. Furthermore, play represents the material stage in the formation of the internal plane of activity, developing the capacity to create systems of typical, generalized images of surrounding objects and phenomena.

Role-play allows children to understand their social experiences deeply. This occurs because, during play, children imagine the roles and behaviors of characters, the objects they require, and the rules they must follow. In play, children model relationships between people, which allow their actions to acquire social meaning (Elkonin, 1987). Through participation in the play, the child is expected to share roles and responsibilities, adhere to rules and resolve conflicts.

Research reveals that thematic social role-play promotes the development of new psychological formations—or neoformations—such as creative imagination, symbolic function, deployed communication, voluntary activity, and reflective thinking (González-Moreno et al., 2014). These psychological formations take root during the child's interactions with adults during play.

From a historical-cultural perspective, the objective of each psychological age is the acquisition of these corresponding new formations. Neoformations are novel aspects of the child's personality that emerge at each stage of development (Elkonin, 1980). Consequently, each neoformation contributes uniquely to the child's psychological growth and personality—for instance, developing the ability to understand others' perspectives and how human relationships work in society.

For Vygotsky (1996), neoformations are elements of the child's psyche that appear as both the goal and the result of each developmental stage. While the nervous system and active participation in society are necessary conditions for their acquisition, they do not inherently guarantee their appearance (Solovieva and Quintanar, 2016). Instead, psychological neoformations arise within the guiding activity, which promotes quality social interactions between the children, adults, and peers (Elkonin, 1980). This guiding activity guarantees psychological development at each stage by subordinating all other activities; conversely, its absence can negatively impact the child's personality and delay the acquisition of new formations (Solovieva and Quintanar, 2016).

During preschool age, thematic social role-play drives the restructuring of a child's personality and development. This activity involves a specific theme or plot, a shared play concept, a sequence of verbal and non-verbal actions, and social interactions. It features multiple interrelated roles governed by cultural behavioral rules, alongside a symbolic component that schematically formalizes these rules. Additionally, the play requires an object or motif that represents a social role, which only gains meaning through interaction with another participant. Consequently, thematic social role-play is a collaborative, joint activity that strengthens emotional bonds and friendships among children.

This activity comprises both external and internal actions. External actions shape the play itself through the social bonds established by the participants, while internal actions develop the play based on the participants' ideas. Guided by adults, these internal components give actions intention, purpose, and meaning.

A child's intentions guide their actions within the play, where meaning is closely linked to motivation and develops through the participants' dialogue about the play's content. For instance, a child play the role of a doctor can help peers acting as patients with imaginary ailments, such as a stomach ache. Here, the intention is to build a connection to help the patients, the purpose is to perform caregiving actions, and the meaning remains personal to each child according to their social and cultural background. This process connects directly to unfolding symbolic actions, enabling children to solve problems and seamlessly link their behavior with other roles.

Likewise, role-play enables the social organization of early childhood learning processes. This experiential framework subsequently reshapes the child's comprehension of their social environment. Within play, the immediate situation is transcended, generating imaginative realms through the reconfiguration of objects and their symbolic meanings (Vygotsky, 1990).

## Psychological formations that develop in the thematic social role-play

The psychological formations—or neoformations—that develop during thematic social role-play, such as reflective thinking, symbolic function, creative imagination, voluntary activity, and advanced communicative activity, are fundamental for academic success during school age and for successful integration into society (González-Moreno et al., 2014). These neoformations establish the essential foundation for a child's transition into formal schooling. Therefore, continuous participation in thematic social role-play during preschool is vital, indicating that play at this stage takes precedence over early academic instruction.

According to Vygotsky (1995), preschool-aged children require play to achieve proper development; thus, play is more critical than formal instruction. This is because the process of play helps children cultivate general skills that lay the groundwork for successful learning. Without this foundation, subsequent instruction is neither as effective nor as comprehensive. Everything must occur at the appropriate time. Consequently, when addressing human development, the principle of timeliness—or the right moment—is crucial. Rather than focusing on speed or quantity, our approach should aim to help children maximize their potential at each developmental stage, thereby securing the groundwork for the next phase.

Learning in preschool specifically functions to contribute to the development of a child's initiative, serving as the basis for formal learning in school age. The learning achieved through play is closely related to the reorganization of psychological processes, or psychological neoformations. In preschool, emphasis is placed on psychological development rather than on the mastery of formal knowledge, which is characteristic of school age.

Through play, children learn to take an interest in communicative interaction, human activity, the temporal sequences of actions, and unexpected turns of events (Bruner, 1988). Thematic-social role-play leads to major changes in the child's needs—such as learning to share, take turns, resolve conflicts peacefully, understand the emotions of others, and comprehend the social world—as well as in their structure of consciousness. The purpose of this activity is social communication with adults and peers, where the main objective is the representation of a specific role. The positive communication dynamic within play becomes a mirror through which children begin to see themselves through the eyes of others, helping them form their own point of view. This positive interaction acts as a mirror, allowing children to see themselves through the eyes of others and build their own perspective. In role-play, children discover new ways to interact and communicate, creating an environment where adults and children learn from each other as equals.

Thematic social role-play allows the development of a child's ability to collaborate, communicate, and understand other participants (Vygotsky, 1995; Tomasello, 2007; Bozhovich, 2014). Social interactions mediated by language promote the internalization of new psychological formations, which in turn expands the child's capabilities. Initially, language serves a communicative function with others; subsequently, it becomes internal language based on communication with oneself (Vygotsky, 1995).

Role-play is a shared activity that enhances a child's abilities, such as solving problems or coping with critical situations after the play has ended. Within role-play, action arises in the imaginary field, contributing to the construction of voluntary intention and the motives of the will (Vygotsky, 1977). This allows for the integration of both shared and individual goals during play.

Furthermore, voluntary activity gives the child the opportunity to set goals and regulate their own behavior. Research indicates that the better a child performs a role in play activity, the higher their level of self-regulation (Bredikyte and Brandisauskiene, 2023). This self-regulation is expressed through interactions with others, the deployment of interrelated actions, and the initiative to initiate and sustain the play activity.

At the interpsychic level, self-regulation skills develop through the utilization of symbolic means—specifically materialized, perceptual, and verbal tools. These means are gradually internalized and transferred into the child's daily activities, becoming integral to their personality development. In this context, materialized means refer to object substitutes (González-Moreno and Solovieva, 2015), perceptual means encompass visual images such as drawings (Solovieva et al., 2016), and verbal means consist of expressions attuned to the communicative situation and play theme (Solovieva and González-Moreno, 2016).

To advance the symbolic function toward complex levels, (Göncü and Gauvain 2012) emphasize the role of intersubjectivity, defined as the construction of shared meaning among individuals. Their research on caregiver-child interactions demonstrates that adults and peers provide critical scaffolding: they guide children's interpretations, embed language and abstract meanings within practical contexts, and assist in negotiating and maintaining rules during symbolic play.

Voluntary behavior is a child's ability to plan, manage, and evaluate their activities intentionally and consciously. It allows the child to overcome dependence on what they perceive in the here and now. Similarly, voluntary activity helps children achieve and maintain goals that match the demands of a situation, monitor and modify their own emotions, maintain and shift attention when necessary, and ignore distractions. It also enables them to understand the meaning of various social interactions, participate in them in a sustained manner, and connect with others by considering what they think and feel in order to act accordingly.

Motivation, self-awareness, and reflection strengthen the development of voluntary activity. Motivation within the role and theme of play contributes to the transformation of a child's behavior. In this way, children begin to learn to regulate their actions and emotions (Richard et al., 2021) by thinking about the consequences these have for others and themselves. This means that a child is able to manage and control their actions while play. The motives formed during play guarantees future interest and curiosity, preparing the child to carry out school study activities in the next psychological stage of development (Solovieva and Quintanar, 2016).

Research by Sukhikh et al. (2023) shows that play develops a child's ability to transfer functions from one object to another, as well as to perceive themselves as an agent of activity. In thematic social role-play, one action replaces another just as one object replaces another (Vygotsky, 1990). This is achieved when the child changes the meaning of an object's use through symbolic actions within imaginary situations. For example, children might imagine that a stick is a magic sword that allows a family of hungry rabbits to find food. This new use of the stick transforms their actions; they now handle it as if it truly were a magic sword. This transformation occurs because imaginary situations arise within the play, which is recreated through a chain of actions (both gestural and verbal) that interconnect with the actions of other roles.

In other words, during play, children—often in collaboration with adults—create an imaginary situation where they assume specific roles, act accordingly, and follow the corresponding rules. The child relies on internal motives to perform these role-based actions, interact with others, and accept the play's rules. Ultimately, this demonstrates how motives guide actions in play.

In play, the relationship between visual and sensory fields focuses on imaginary ideas rather than a realistic perception of the environment. The meaning of objects is subordinated to the dynamics of play. Consequently, children create imaginary situations and build social rules for interaction. Vygotsky (1977) argues that play cannot exist without rules; only actions that occur in accordance with these rules are acceptable within the play context (e.g., waiting for one's turn to speak because not everyone can speak at the same time).

Research highlights the role of play in children's socio-emotional learning (Walker et al., 2020) and in the development of self-regulation (Bredikyte and Hakkarainen, 2017). Through these activities, children learn to process complex emotions—such as fear, joy, and frustration—by navigating imaginary scenarios within safe spaces. Furthermore, play fosters empathy and essential social skills, including cooperation and conflict resolution, which are subsequently transferred to other areas of their lives. Consequently, role-play creates the zone of proximal development; by assuming complex roles within guided play environments, children can execute individual and social actions that they are not yet capable of performing in real life. This play generates co-construction dynamics that expand the child's cognitive capabilities in a motivating way.

## Conceptual model of thematic social role-play

In role-play, the motive (or object) is the enactment of a social role that only acquires meaning through the presence of another participant within the same imaginary situation. Therefore, this type of play cannot be considered a purely individual, free, or spontaneous activity. In fact, free play lacks a stable motive, as it constantly shifts from one object to another, from person to person, and from one expression to another, without requiring a third party as a constant element.

Through play, children develop affective and verbal communication; their linguistic expression unfold and takes on a voluntary character, much like their other psychological functions. The need to enact specific roles implies that children simultaneously submit to cultural rules of behavior; in other words, their conduct loses its spontaneous and immediate nature (Vygotsky, 1996). Thus, engaging in play allows them to enter an imaginary world distinguished by its emotional significance, thereby optimizing the psychological development characteristic of this stage.

The structure of play activity presupposes the existence of a need, a motive, a goal, and the operations required to carry it out. Its content involves various interrelated roles, where adopting a role implies adherence to cultural norms. Beyond these roles, it is possible to analyze the theme or plot of the play, as well as sequences of verbal and non-verbal actions. Also notable is the presence of a symbolic component in thematic role-play, which involves the regulation and schematic formalization of certain rules.

This symbolic development takes on immense significance during the preschool years. According to Davydov (2008), the symbolic function allows one thing to be identified through another, substituting real objects with signs or mental representations. This process lays the groundwork for abstract thought and the internalization of culture; thus, the substitute object becomes an objectification of the original object's characteristics or functions, acting as a regulator of action and behavior.

These characteristics of play allow children not only to internalize the play and respect its structured rules but also to use symbolic means as tools for behavioral self-regulation (e.g., through the sequential ordering of turns in a veterinary clinic scenario). Consequently, the external symbols employed during play reflect the essence and general patterns of various communicative situations, such as attending to customers on a first-come, first-served basis, repairing watches according to their models, or constructing a city based on prior planning.

During the preschool years, the symbolic function not only enables children to apprehend the world's diverse characteristics but also fosters a selective and organized perception of reality. In this context, Ilyenkov (2009) maintains that the symbolic realm originates within the imagination during the preschool stage, laying the groundwork for attaining higher levels of symbolization—linked to abstract conceptual modeling operations—during the school-age years. The creation and use of signs and symbols constitute the primary mechanism of human cultural development, the behavioral structure of which rests firmly upon mediated activity (Vygotsky, 1995).

### Ontological definition of role-play

Thematic social role-play is defined as a social, rule-based, and collective activity that differs from free play by possessing a stable motive: the enactment of a social role. While it requires structural boundaries rather than pure spontaneity, its meaning emerges exclusively from interaction with another participant—or the system itself—within a shared imaginary situation.

A play begins with choosing a theme. The goal is for the children to define the aspect of reality or the social situation they will represent (e.g., a restaurant, a veterinary clinic, or a toy shop). With the teacher's help, the children identify the social roles involved in that situation. For instance, if the theme is a restaurant, the roles would include cooks, waiters, customers, a manager, and a cashier.

The distribution of roles can be done according to each child's wishes, but should always take into account their particular characteristics and needs. The play must guarantee that roles can be changed in subsequent play sessions with the same theme. In this way, children have the opportunity to experience different positions in social relationships. Each role requires a specific type of behavior and action, which changes as the role-play becomes more complex. For example, if a child is aggressive toward others because their immediate desires are not met, these attitudes are later transformed into more compassionate actions toward others and themselves. Ultimately, the role determines the attitude and actions of the children. The numbers of roles, as well as the depth and complexity of the communicative actions between participants, vary depending on the social situations represented. When organizing play interventions, several criteria must be met for successful adult participation: a shared play theme that captivates all participants; active adult participation through role-taking; the adult's emotional engagement in the play to foster the children's own emotional involvement; and dialogic communication between the adults and children.

Next, the characteristics of the roles are identified together, the materials to be used are chosen, the space is organized using symbolic means, and the play begins. Finally, there is a reflection on the play and the actions of the roles, and new proposals are made.

### Structural components of the model

The architecture of the role-play activity comprises four interconnected levels, complemented by an essential feedback mechanism:

- Need and motive: the driving force of the activity. The primary motive is the embodiment of a social role, which intrinsically requires a co-participant to serve as a constant social anchor.
- Roles and cultural norms: by adopting a specific role (e.g., physician, firefighter, painter, paleontologist), the child voluntarily submits to the cultural and behavioral rules associated with that function. This submission suppresses immediate, purely impulsive behavior.
- Plot and theme: the overarching narrative that guides the sequence of the participants' verbal and non-verbal actions. This narrative is co-constructed through ongoing dialogue between the adult and the peer group.
- Symbolic component: the concrete actions that materialize the play activity. This includes the formalization of rules and the deployment of symbolic functions, where real objects are replaced by semiotic or conceptual substitutes (the substitute object serving as an objectification of the original).
- Feedback: feedback should be precise, action-oriented, and delivered within an emotionally safe environment. Before receiving feedback, children need to feel that the teacher is an ally—someone seeking to help them play and learn better—rather than someone who judges or punishes them. Therefore, it is crucial to avoid abruptly breaking the magic of the play or exposing a child in front of their peers using a reproachful tone. Ideally, teachers should wait for a pause in the play or the end of the activity to talk and reflect. It is also key to be specific about what was done well in order to strengthen self-efficacy and ensure the child knows exactly what strengths to build upon. For instance, instead of saying, “You played veterinarian so well!,” it is better to offer a precise assessment: “I loved how you checked the stuffed dog's ears with your flashlight and how you calmed the owner down by telling them not to worry.” Similarly, rather than diagnosing or criticizing a mistake (“You forgot to collect payment”), feedback should be framed as a guidepost indicating the next step. Feedback must focus on what the character is doing in the scene, never on the child's personal traits. For example, instead of focusing on the person (“You're very absent-minded; you forgot to use the money”), one might suggest an action: “Next time you're the cashier, remember to ask the customer for the coins before handing over the bag. Shall we do a practice run together?”

### Relationship dynamics (How the model operates)

- Self-regulation through imagination: entering an imaginary world rich in emotional significance compels children to subordinate their immediate desires to social norms. This mechanism transforms impulsive behavior into voluntary, self-regulated conduct.
- Social interdependence: roles are inherently relational; Role “A” only acquires meaning in coexistence with Role “B.” Consequently, the stability of the play activity depends on the continuous, responsive presence of the other.
- Semiotic substitution: the use of a substitute object (e.g., using a pencil to represent a syringe) acts as a cultural mediator. This is not merely a material replacement, but a mental identification process that transfers the functional meaning of a real object onto a symbolic sign.

### Outcomes in psychological development

The conceptual model concludes that role-play serves as the leading activity for development during the preschool years, fostering:

- Linguistic and affective development: the expansion of verbal communication, which begins to take on a voluntary, self-regulated character.
- Internalization of culture: the appropriation of the dynamics, roles, and social norms of the adult world.
- Genesis of symbolic thought: a transition from direct action with physical objects to the manipulation of mental representations, facilitated by symbolic function.

Box 1 presents an illustrative example of how the role-play conceptual model is developed. Active actions are identified within a space that is both emotionally safe and cognitively challenging for both adults and children.

Box 1 **Example of how to conduct the role-play.**

•**Age group:** 5–6 years old

•**Theme:** the lost puppy (Empathy and Problem-Solving)

•**Objective:** to develop collaborative problem-solving through imaginative play.

**1. The setup (Introduction)**

The adult gathers the children in a circle and introduces a prop—a small plush dog.

“Class, look who I found sitting by our classroom door. This is Milo. He looks a bit sad because he went for a walk in the imaginary forest and cannot find his way home. Can we help him?”

**2. Role assignment**

Instead of rigid scripts, children choose general roles to drive the narrative:

•The search party: children who look for clues.

•The animal helpers: children play forest animals (birds, squirrels) who can give clues.

•The comfort team: children who build a temporary shelter or bed for Milo.

**3. The guided narrative (Action)**

The adult acts as the narrator, guiding the plot while letting children make choices. Adult language makes it possible to regulate children's actions.

•*Scenario 1:* asking the forest animals

Narrator: “We have arrived at the edge of the Whispering Woods. Oh look, there are some friendly birds in the trees! Search party, what should we ask them?”

Child (Bird): “Tweet! I saw him walking by the big blue river!”

•*Scenario 2:* crossing the obstacle Narrator: “The river is too wide to jump over. How can we get across to find Milo's house?”

Children: (Suggest building a bridge with foam blocks or pretending to row a giant boat).

**4. The resolution**

The children successfully navigate the obstacles and find Milo's doghouse (a decorated cardboard box).

“We made it! Milo is safely back home. Look, his tail is wagging! How do you think Milo feels now that he is home?”

**5. Reflection & debrief**

The activity concludes with a brief circle-time discussion:

•What was your favorite part of the rescue?

•How did we work together to solve the problem?

## Relationships between thematic social role-play, creative imagination and symbolic thinking

For Lev Vygotsky, creative imagination and symbolic thought are higher psychological processes that emerge from social interaction and the use of cultural tools. These include external means, such as language, which transform the environment, and internal means, like ideas, which regulate and transform human behavior itself. Rather than being innate, creative imagination arises from the experiences accumulated through a child's leading activity. The brain does not merely retain past information; it recombines experiences to construct something entirely new. Consequently, the foundation of creative imagination lies in human activity, which enables both the projection of the future and the modification of the present through social interactions like role-play.

In other words, creative imagination transforms the present from a mere complex of active impressions or a synthesis of past experiences into a tangible possibility for future action. This process emerges when children detach from concrete objects to represent them through actions and words. For instance, when a child imagines a box is a spaceship, they combine personal or observed experiences during play to construct new realities based on their interests and needs. Similarly, symbolic thought arises from semiotic mediation involving symbols and signs, particularly language. It is conscious, voluntary, and self-regulated. Because its origin is social, historical, and culturally mediated rather than biological or spontaneous, symbolic thought does not develop in isolation. Instead, it is first co-constructed through interaction with others (the interpsychological plane) and subsequently internalized by the individual (the intrapsychological plane).

There is a close relationship between intellectual development and symbolic development (Vygotsky, 2001). Research indicates that the lower the child's overall developmental level, the less development is shown in their play activity (Salmina, 2019). Any deficit in intellectual activity is evident in symbolic function, as well as in verbal, artistic and playful activities (Vygotsky, 2001). For example, children with cognitive deficits have difficulty differentiating real actions from imaginary and symbolic actions; their use of toys is stereotyped, verbal accompaniment is very limited or absent, and there is a lack of initiative to propose creative actions that allow them to establish links with other roles (Salmina, 2019).

Creative imagination and symbolic thinking enhance children's cognitive, emotional, and social development. Imagination allows children to create external means of understanding a current situation and its relationship to imagined situations. Creative imagination is an important product of play in the development of a child's personality (Davydov, 1996). Play begins when the child manipulates objects, and then acts out brief episodes of daily life, and finally, imaginative role-play emerges (Elkonin, 1980). Role-play is inherently imaginative. This is expressed when children adopt roles different from their own, which requires “stepping outside of oneself,” managing emotions, and making decisions from the perspective of the character being portrayed. In this process, the child uses various symbolic means that allow them to transform reality. For example, when a child uses a box, they imagine that it is a boat and perform the corresponding actions as if it were real.

The symbolic function is the cornerstone of creative imagination, enabling children to construct new realities and possible worlds. In social interactions, for instance, it fosters greater compassion and solidarity. Furthermore, this cognitive ability enhances the understanding of both one's own emotions and those of others.

Developing this function is highly driven by thematic social role-play, where the creation of imaginary worlds plays a vital role. It manifests when a child uses one object as a substitute for another, makes a gesture to represent an action, or employs language to build an imaginary scenario. It is also expressed when they assume a specific role and use verbal expressions to forge connections with others.

In thematic social role-play, object-directed actions are perfected and generalized, serving as a basis for acquiring more complex symbolic actions (Elkonin, 1980). The development of these actions generates both the image and the mental representation of the activity (Elkonin, 1995). Throughout this process, thought is expressed through gestures, actions, and verbal communication.

This symbolic function manifests in play, verbal communication, and artistic activities like drawing (Salmina, 2019). Within play, symbolic tools help organize and regulate a child's behavior. This function fosters symbolic thought—the cognitive ability to mentally represent objects, people, ideas, or emotions through absent symbols (words, images, or gestures). Furthermore, the symbolic function enables the use of various external means to formalize and guide play activities. A specific sign demands a corresponding behavior that is not arbitrary but aligned with the sign's meaning. For instance, in the themed play “The train,” children subordinate their behavior to traffic signals, which effectively establishes the rules of engagement.

During play, children establish rules based on the situation. These rules effectively regulate their behavior. While adhering to them provides a sense of satisfaction, the greatest gratification comes from resisting immediate impulses and following rules of their own making, which ultimately leads to an illusory satisfaction of their needs.

Similarly, play creates an internal tension; for instance, a child may cry while play the role of a patient in pain, yet simultaneously feel happy as a participant (Vygotsky, 2005). Because the child's actions are confined to the role-play, they can be more consciously controlled than in real life. Consequently, developing a character creates psychological tools—such as emotional regulation—that are essential for transforming a child's actual character and social interactions.

Role-play also creates a zone of proximal development for children and is highly effective in stimulating the development of self-regulation (Vygotsky, 2016). This implies that play must possess specific characteristics to foster a child's optimal development. This aligns with mature forms of play, where children fulfill their roles effectively, establish respectful social interactions, subordinate their personal desires to the rules of the play and reflect on their own behavior.

Research indicates that thematic social role-play evolves from simpler forms into complex, mature stages (González-Moreno, 2015). The defining feature of mature thematic social role-play is the ability to develop shared ideas and co-construct a storyline that sustains itself over time. Within this framework, adults and children collaboratively create and inhabit imaginary worlds, solving problems through mutually developed ideas.

In play, the child sees their own actions for the first time because they are simultaneously themselves and another person (Elkonin, 1980). Assuming a social role in play is a complex form of symbolic function development. This implies that the child adopts a role and assumes its rules and attitudes through their actions. By developing this skill, the child learns to know, accept, and improve themselves. Thus, the child's actions in play are interrelated with those of other roles in imaginary situations. There are also conscious intentions, motives, and the construction of a plan. Consequently, children experience the problem as they solve it (Vygotsky, 1977).

The performance of a role becomes the motive for the play activity. After obtaining the desired role, the child acts according to its rules. In play, the rule directs the actions of the role; the child regulates the actions of others and, subsequently, self-regulates. By assuming the role, the child supervises and controls their own behavior through its correct execution.

Play unites children through these roles, which require them to follow the rules under adult guidance through reflective questions or symbolic gestures. This prevents children from abandoning the activity prematurely. Thus, the participants are immersed in an imaginary situation in which they begin to regulate their behavior. If children are interested in a play activity, they will mobilize all their efforts and attitudes to develop it in depth. This form of participation allows children to behave above their chronological age (Vygotsky, 2016).

From the point of view of psychological development, child-initiated play is an activity in which their interests, motives, and abilities are revealed. This highlights another characteristic of mature play: initiative. By creating meaningful joint play, children mobilize their full potential and often go beyond their everyday behavior. For instance, a child who has difficulty regulating their behavior learns self-regulation through play by assuming a social role. In the case of play doctor, assuming this role transforms their actions and expressions, making them more reflective and compassionate, as their focus shifts to caring for and helping others.

In play, children have the opportunity to create something new: a movement, an object, an idea, a sense, or a meaning. A child who participates in this creative process develops new psychological formations that lead to a qualitatively new level of functioning. For example, in restaurant-themed play, a child pretending to be a chef creates food using figures to serve to their diners. This process naturally involves countless social interactions.

The numerous interaction experiences that children gain with peers and adults during thematic social role-play help them begin to monitor their own behavior and activities. In play, children often act against their immediate desires and test the limits of their willpower. Ultimately, their motivation and role performance transform their behavior (Smirnova and Ryabkova, 2010).

Therefore, professional development for both pre-service and practicing preschool teachers should focus on finding ways to facilitate and support the development of imagination and symbolic thinking in all children. The actions that take place within the imaginary context of thematic social role-play acquire new meaning and teach children to guide their own behavior (Vygotsky, 1995). The child progresses from a materialized phase—where realistic toys are needed to think and play—toward a level of abstract, symbolic, and playful maturity. At this higher level, language becomes the primary psychological tool that sustains imagination and thought. Table 1 presents the indicators of the development of creative imagination and symbolic thinking. These indicators are part of the theoretical proposal of this article. The value of these indicators lies in the creation of personal meaning. This means that the child assigns emotional value or a unique purpose to play situations, going beyond the mere imitation of adult actions in real life.

**Table 1:** Indicators of the development of creative imagination and symbolic thinking.

Creative imagination (Transforms representations of ideas into new creations)	Symbolic thought (Representing ideas using materialized, perceptual, and verbal means)
•Possibility to develop, expand and detail an idea, increasing its complexity and perfecting it in the play.	•Possibility of performing sequences of actions with objects as substitutes for others, which helps in role interpretation. •Objects are replaced by imaginary objects or symbolic content in the interactions between roles.
•Possibility of considering different ideas and perspectives to solve a problem that arises in the play.	•Possibility of using symbolic means on the graphic plane to regulate the behavior of other roles and one's own role.
•Possibility of communication with the other roles about their imaginary actions.	•The possibility of creating imaginary situations through words that enrich the actions.

## The adult's guidance in role-play

The adult guides the development of the children's joint activity and provides individual support to those who need it (Hakkarainen et al., 2013). During orientation within the play situation, the mental image of the action—which subsequently guides the child's execution—becomes integrated. The formation of any action simultaneously leads to the formation of the image of its object; thus, the characteristics of that image largely determine the characteristics of the action itself (Galperin, 2001). Orientation within a situation involves a series of steps and transformations, progressing from external, fully deployed actions to internal, condensed ones (Quintanar and Solovieva, 2019). Furthermore, the degree of an action's automation indicates the level of conscious, directed control involved (Galperin, 2001). This automation develops through the reflective use of various media, including materialized, perceptual and verbal tools.

The guidance received during play is initially external, mediated by language and adult models, until the child develops initiative and autonomy in carrying out material, materialized, perceptual, and verbal actions. In thematic social role-play, the performance of object and symbolic actions—through movements and verbal expressions—enables an imaginary configuration of the play situation.

Research on thematic social role-play in preschool suggests that teachers who continuously implement this activity introduce a creative dimension to their professional work (González-Moreno, 2016). This allows them to develop educational practices that consider both children's individual needs and their cultural contexts.

The teacher must be able to observe, guide, support, and evaluate the quality of children's play while actively participating as a playmate. To do this effectively, the adult needs to observe the children's actions, their transitions between activities, and the interactions among all participants.

Teacher involvement is most beneficial when it introduces new play strategies that expand and enrich children's initiatives and ideas. For example, adults can encourage children to sustain their play by using strategies that help them imagine how to advance the narrative and develop their roles.

Another key pedagogical aspect is the teacher's ability to perceive children's interests during play, which can then be used to plan and organize future interactive sessions. When the teacher offers meaningful, engaging strategies that help children connect actions to solve problems, it inspires and enriches the experience. This progression—moving from teacher-initiated play to play where initiatives arise directly from the children—is the very basis of its transformation.

Children learn to play under adult guidance when it is explicit and clear. For instance, this occurs when adults help organize the play environment by defining the roles, actions, and objects involved. As play sessions progress, children become increasingly emotionally invested. This deep emotional involvement allows children to internalize and represent emotions consciously, strengthening their bond with both the social environment and the experiential activity through dialogic communicative interactions.

These dialogic communicative interactions between the adult and the children emerge directly from their joint activity during play. Initially, the adult proposes the play framework, which includes the theme, roles, and actions. However, as children become more emotionally engaged, their own initiatives and proposals begin to surface. Consequently, each play experience enables children to participate more actively, drives the development of more complex play ideas, and extends the overall duration of their play.

The adult guides the thematic social role-play activity through dialogic conversation. Some strategies that the adult uses include verbal and non-verbal dialogue (such as eye contact, gestures, body language, and pauses) and the expansion or strengthening of the child's experience and skills through the zone of proximal development. When the adult identifies a child's attempts to be independent in play, they gradually reduce their support. This scaffolding transforms the child's actions, making them more responsible and self-regulating in the bonds they establish with others and with themselves. A child with emotional self-regulation makes decisions in play by thinking before acting and recognizing their emotions instead of reacting impulsively. Adult guidance also allows for the modeling of emotional regulation strategies to help children join and remain in the play. For example, the adult demonstrates within the play how to manage emotional intensity by negotiating conflicts and respecting turns.

This proposal clearly distinguishes between direct control over children's play and the provision of supportive guidance. We often confuse intervention with control. However, while rigid control extinguishes the spark of a child's autonomy, guidance fans the flames. Guiding without imposing means being present to enrich play, validate emotions, and open new avenues for exploration—ensuring that play remains what it is meant to be: an activity that fosters personal growth and open-ended experience.

In this way, the adult's guidance allows the play to transform into a level of reflective initiative, creative imagination, and symbolic verbal actions. This transformation occurs because the child becomes emotionally connected to the theme of the play and the actions they perform. Furthermore, the child realizes that there is something they already know and something new in the play, which transforms their motivation by giving it meaning. Thus, the child finds that their actions are relevant to themselves and to others.

At first, the child continues the dialogue proposed by the adult and accepts their initiatives. Then, the child initiates the dialogue and maintains it with their playmates. The child also introduces new initiatives regarding the play's content. They use facial expressions and gestures that reveal the emotions linking them to the play. The child's emotions are related to the meaning of the situations and actions that occur within them (Zaporozhets, 2002).

When children become emotionally engaged in teacher-led role-play activities, they often want to share these experiences with their parents. This desire is a strong indicator of healthy psychological development.

To support this growth, parents should be encouraged to actively join their child's imaginative plays at home—whether acting as a doctor for a teddy bear or the captain of a cardboard ship. While play, adults should show genuine emotional engagement, setting aside any fear of appearing childish.

It is also crucial to advise parents against using playtime as a disguised lesson. Instead of asking academic questions like, “What color is this block?” they should focus on solving problems within the narrative of the play (e.g. “Oh no! The tower bridge is too short; what material from the magic forest can we use to make it longer?”).

Furthermore, parents can be invited to bring stories to life after reading aloud, rather than simply closing the book. Families can create shared settings at home—using everyday items like sheets, boxes, or cushions—to continue the narrative. Finally, parents can help gather recycled or natural materials. Through this, they learn that the true value of a toy lies not in its finished form, but in the collaborative ability to assign a “superpower” or new function to an ordinary object during play.

## How do children learn to play and what are the central elements of this learning process?

In thematic social role-play, the zone of proximal development is transformed into a special form of jointly constructed interaction, where the adult's action specifically stimulates and supports the child's initiative (Zuckerman, 2007). For this reason, cultivating a high-quality social bond and interaction between adults and children is necessary in play. In Table 2, we present a theoretical proposal demonstrating that communication fosters an emotional bond among play participants. For example, the ability of adults to take on interesting and challenging roles becomes a way of modeling actions. These actions are then taken up by children in play, where they are transformed and enriched with symbolic actions. The keys to this communication process are: affective attunement, where participants read and respond to each other's gestures and emotions in real time; recognition of the other, where playmates complement each other's contributions; and the use of expressions, postures, and words to sustain the shared fiction, which regulates coexistence within the play.

**Table 2:** Content of communicative interactions in the play.

Elements of communicative interactions	Description	Indicator
Alternating conversational turns	•Communication is active and reciprocal.	•Participants alternate between the roles of speaker and listener. •The conversation is structured through speaking turns, sometimes with interruptions. •Alternating shifts enables cooperation between roles during the execution of actions.
Dialogical nature of interactions	•Interactions are characterized by dialogue, active listening, and communicative exchange.	•The children accept the previous action (materialized, perceptual, verbal, non-verbal) and add their own action during interactions in play.
Dynamic exchange of information regarding the rules	•It refers to the interactive feedback between participants regarding the rules.	•Children propose rules and express them verbally to regulate their actions. •The children accept the rules of the play.
Use of verbal and non-verbal signs	•It includes words, gestures, tone of voice, posture, and use of space	•sVerbal and non-verbal signs contribute to the negotiation of meanings in the play.
Intentionality	•The ability to understand oneself and others as agents who act with their own goals, desires, and mental states in their communicative interactions.	•The participants in the play seek to express needs, desires, or achieve a specific goal.
Active cooperation	•There is collaboration between children and adults in the interactions that occur during play so that it progresses toward complex forms.	•The children exchange ideas and use symbolic means to achieve a common goal.
Initiative	•In the interactions that occur during play, the adult encourages the child's initiative to develop the ideas that arise together.	•Children express their ideas verbally and/or through the use of materialized or perceptual signs.

The teacher needs to synchronize social interactions with children's emotional expressions. When an adult matches the nonverbal expression of a child's underlying emotions, the child can experience the adult's empathy and better regulate those emotions during play. Emotional communication also helps adults become aware of and deepen their own social and emotional experiences. In other words, children can learn to see themselves reflected in the emotions of others.

Teachers also need to pay attention to their own nonverbal communication and that of the children. These forms of communication are key signals that help educators understand what often cannot be expressed in words. In addition, children pick up on the subtleties of body language and facial expressions, which indicate whether others are comfortable or annoyed. For example, eye contact demonstrates interest and a desire to engage further in the play. If teachers identify any signs of discomfort during play, they can adjust their strategy to ensure all children feel safe.

Research reveals that when adults take a genuine interest in the play experience and listen attentively, children feel confident enough to actively participate in interactions and the unfolding conversation.

The content of the play is enriched by new ideas that children develop during interactive conversations with adults and peers. Likewise, the experiences children gain in each play activity shape the emergence of new ideas, which evolve through the recreation of novel imaginary situations and the introduction of role-specific rules. Previous research shows that the progression of role-play requires the presence of several elements, as shown in Table 3 (Solovieva and González-Moreno, 2017). For example, topics are initially proposed by the adult; later, children—on their own initiative—propose new themes or revisit previous ones with new elements. This information represents a conceptual synthesis.

**Table 3:** Content of the thematic social role-play activity.

Elements of the activity	Description	Indicator
Social situation of development	•Type of relationship between the child and society.	•The teacher continuously generates thematic social role-play activities for the group of children.
Topic proposal	•They stem from the historical and social conditions that surround children.	•The adult suggests the play theme. •The children choose the topic together with the adult. •The children choose the theme of the play on their own initiative.
Choice of roles	•It can be done randomly using external means or according to the children's opinion.	•Meets the objective. •Regulates behavior through their own verbal language or that of others. •Takes the initiative to set objectives. •Respects the objectives of others.
Content analysis	•Together, the adult and the children reflect on the execution of the play.	•Use questions such as: of what we did, what did we like? What didn't we like? Did what we did work? Which strategies were helpful? Which ones would we use again? If we had to do the process again, would we do anything differently? What and why? •Possibility of considering several courses of action to make a decision. •Possibility of resolving conflict situations. •Reflection on one's own actions and the actions of others.

Learning through play is based on the relationship between the imaginary and the real (Hakkarainen, 2009). Understanding the imaginary situation—where children consciously experience and reflect upon their context by relating reality to the social sphere—leads to a reorganization of their thinking. This cognitive reorganization modifies their understanding of social interactions by reshaping the relationship between the child and their social environment. Consequently, the development of creative imagination and symbolic thought fosters intersubjectivity. Intersubjectivity is defined as the shared, reciprocal experience between the teacher and the children, as well as among the children themselves; it allows children to realize that they can influence others and vice versa. For example, children feel valued when they are listened to and when they see their ideas translated into actions that drive the play forward.

In this manner, social role-play progresses from the use of realistic objects to object substitutes, accompanied by an increase in initiative, the proposal of new play scenarios, and ultimately, narrative social role-play. Previous research has identified indicators of the zone of proximal development in role-play (González-Moreno, 2016) that demonstrate a transformation in the child's development (see Table 4). These serve as illustrative examples.

**Table 4:** Development indicators in the stages of role-play.

Stages	Description	Indicator of the zone of proximal development
Social role-play with the use of objects	•The play's content consists of actions with objects.	•To enact the role, children's actions are directed toward the use of objects.
Social role-play with the use of objects and object substitutes in various types of situations	•The content of the play consists of actions with objects and object substitutes in interaction with other roles.	•Children's actions exhibit greater variety when they interact with individuals in different roles.
Social role-play with object substitutes	•The play consists of actions using object substitutes.	•To represent a role, children direct their actions toward using object substitutes.
Social role-play with increased initiative and proposal of new plays	•Play content consists of roles defined by the participants' actions. •The children coordinate their actions, socially negotiating the topic, roles, and actions.	•Peer interaction: children show interest in their peers, observe one another, share goal orientations, negotiate, and co-create meanings. •Action variety: their actions and behaviors become more varied and widespread. •Role immersion: children fully immerse themselves in and embody the roles they play. •Rule awareness: they actively experience and navigate the implicit rules of their chosen roles. •Emotional complexity: they experience conflicting emotions simultaneously—such as happiness and sadness—and consciously reflect on their emotional state.
Narrative social role-play	•Through narrative role-play, children jointly construct a story, navigating various imaginary situations to solve a problem.	•Children take the initiative in creating collaborative stories and solving problems that arise during play.

Box 2 illustrates the stages of social role-play among a group of preschool children. As these illustrative play sessions progress, the children develop essential social skills such as empathy, communication, trust, and collaborative problem-solving. They also learn to value their peers' diverse ideas and reach agreements. This mutual understanding motivates them to actively participate and interact, fostering a harmonious environment that contributes to their mental well-being at school.

Box 2 **Examples of the stages of thematic social role-play.**

**Table:** 

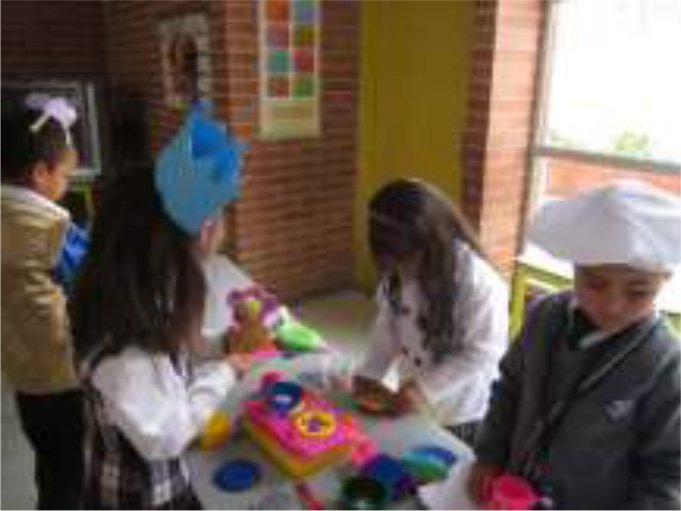 Stage of social role-play with the use of objects. Theme: “The restaurant” The children use various props, including a chef's hat and a queen's crown, as well as themed toys that correspond to the play.	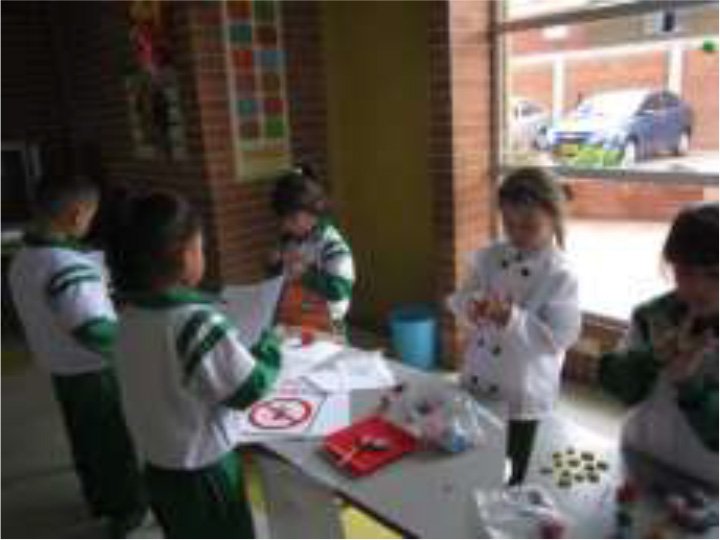 Stage of social role-play using substitute objects. Theme: “The fruit shop” The children work together to create a fruit shop menu using tokens and modeling clay. They negotiate and agree on what to include in it.
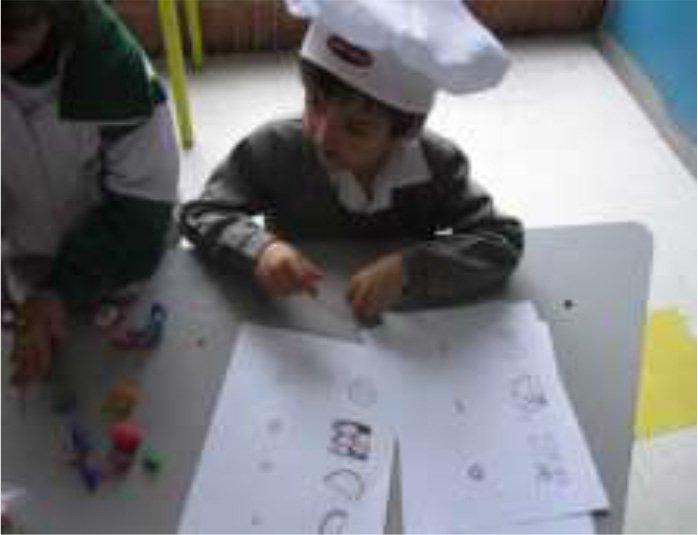 Stage of social role-play with the use of object substitutes at the symbolic-perceptual level. Theme: “The restaurant” The children create symbols of the foods they will prepare, using modeling clay to make the items they will sell in the restaurant.	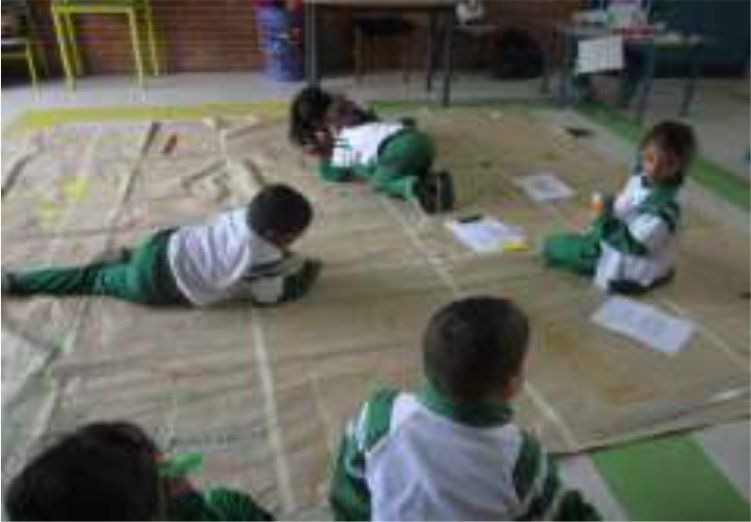 Stage of social role-play with increased initiative and the suggestion of new plays. Theme: “City designers” In this collaborative activity, children work together to bring their dream city to life through drawing.

Furthermore, the thematic toys employed include objects that facilitate the development of working memory skills—specifically for organizing actions at a perceptual-symbolic level, as proposed by Gavrilova et al. (2025). Working memory is crucial for preschoolers, as it enables them to retain information—an essential aspect of learning, following complex instructions, language comprehension, and solving problems during play.

## Example of a narrative role-play

The following example is presented as an illustrative anecdote: the Farm Rescue. The play began with a message: “A flood destroyed my aunt's animal farm. Two rabbits and three sheep are trapped. We need your help to rescue them because they fell into the sea.”

Once the teacher read the letter, a girl suggested creating a boat out of cardboard boxes to sail across the water and look for the animals. Several children quickly joined the initiative. First, they drew a model ship together. Then, one child brought the largest box from the classroom, while others gathered scissors and tape.

The teacher helped cut the box. Together, they shaped the base, formed the bow by joining the sides, secured the structure with tape, and added masts.

Then, the teacher invited the children to get on the boat. A boy chose to be the captain. He began to move his hands from side to side as if he had the steering wheel and was driving it. Another child used a circular piece of cardboard as if it were binoculars to help in the search. Another child was waving his hands, making gestures as if he had a stick to make the boat move forward. Another child chose to be the doctor to rescue any injured animals. Some children were the rabbits and others were the wounded sheep. A girl joined the doctor as a nurse to heal the wounds of rabbits and sheep. A child brought food for the animals. When the ship reached an island, the boy with the binoculars shouted loudly: “I see them,” “I see them,” “there are the animals.” At that moment, everyone got off the boat. The doctor attended to the injured animals. The nurse helped him. Then, a child gave them food.

Then the teacher asked, “How do we get the animals back?” A child suggested, “We have to put them on the boat.” Another child placed small balls on the floor as if they were food to make a path and guide the animals to the boat. The play continued. The rescued animals returned to the farm. A boy was a firefighter and helped clean the farm. The play ended with all participants celebrating the rescue.

In this play, there was a sequence of dialogic interactions between the teacher and the children through the creation of imaginary situations. The children took several initiatives to express their ideas through actions and within the resulting conversation (Table 2). These are illustrative examples.

In social role-play narratives, children and adults co-create a shared idea, constructing a playful narrative that fosters active participation. Similarly, in narrative play, children generate new interpretations of stories—such as experiencing a flood, rescuing animals, guiding them home, and rebuilding their destroyed dwellings—modifying them to make them meaningful as they tell their own stories (Veresov et al., 2025). They also enact events through physical actions and expressions. As children explore historical themes that spark their interest, the adult encourages them by offering various play options, such as devising different ways to build a rescue boat or the animals' homes. Consequently, play enables adults to better understand the children—recognizing what brings them joy, surprise, or sadness within the story—while expanding their own problem-solving capabilities during the activity.

The children demonstrated significant agency by taking the initiative to express their ideas, bridging the gap between physical actions and verbal communication (Box 3). These behaviors serve as illustrative examples of their learning processes. They demonstrate that children do not rely on a single mode of expression, but rather reinforce their spoken words through concrete actions. In narrative role-play, children create perceptual symbols to define the play space. They enjoy this process because it allows them to construct symbols collaboratively to sustain the play (see Box 4 for illustrative examples).

Box 3 **Example of a conversation in a narrative role-play.**

**Boy:** this flood was bad because it destroyed the animals' house on the farm.

**Girl:** *makes sad gestures*.

**Girl:** what can we do?

**Boy:** we can build a house.

**Girl:** but they're big animals.

**Girl:** I don't think they'll fit in a house.

**Boy:** so what do we do?

**Boy:** we could make a house for the sheep and another for the rabbits.

**Girl:** yes, let's make them.

**Teacher:** what could we use to make the houses?

**Boy:** look, there are cardboard boxes.

**Girl:** yes, we can cut them and make houses for these little animals.

Box 4 **Example of perceptual symbols created by children.**

**Table:** 

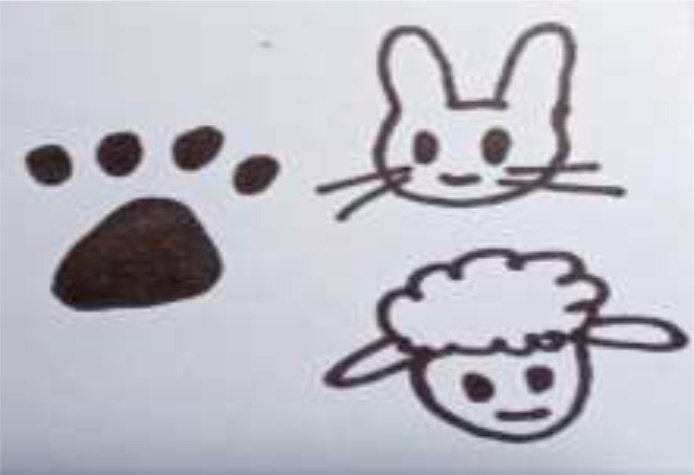 Symbol of rescue: rabbit and sheep.	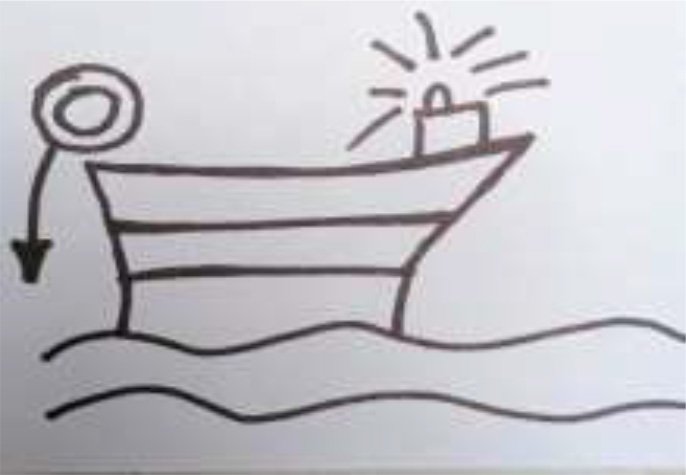 Symbol of a rescue ship. This ship has lights so others can see that we are rescuers.

Teachers can also involve families, as children often return home from school-based play sessions eager to share the play and how they resolved various situations. At home, they invite parents, siblings, and extended family to join in, extending the play into the domestic environment. At this stage, the depth and skill with which a child enacts a role during play directly correlates with higher levels of emotional and social self-regulation in daily life.

## Discussion

This article addresses the complex relationship between thematic social role-play, creative imagination, symbolic thought, and child development from a historical-cultural perspective. The study highlights the cognitive and emotional nature of these developments in children to contribute to the development of communication and social interaction skills. Play exercises not only the mind but also emotions. By assuming the role of a doctor, a mother, or a firefighter, children experience complex emotions, learn to delay immediate gratification, and voluntarily submit to the rules of the role, thereby developing emotional self-regulation. For example, the use of dialogue throughout social role-play indicates that children use language independently to guide their actions and those of others, in accordance with the primary objective of play: to establish social relationships.

The development of creative imagination and symbolic thought constitutes a series of qualitative changes in each child within the context of play. The psychological connections linking children to reality and to other people are not direct. In this activity, children think, act, and communicate using various psychological tools that guide their psyche. The process of mediation is a fundamental condition of the human psyche. Bakhurst (2007) described the importance of cultural mediation for human thought, highlighting five aspects: (1) cultural mediation is a uniquely human psychological capacity; (2) it creates the possibility of self-awareness; (3) it is a condition for creative thought; (4) it is a condition for the development of subjectivity; and (5) it creates a unity between the human psyche and the world.

Unlike theories that view play as a biological instinct or a purely individual activity, the historical-cultural perspective maintains that role-play is social in nature. Children do not invent these dynamics out of thin air; rather, they recreate the activities and relationships of the adults in their culture. Role-play allows for the development of intersubjectivity and subjectivity. Intersubjectivity—the capacity to share emotions, intentions, and understanding with another person—develops during play through a dynamic process of attunement and co-creation. This encounter with the other takes place through mutual eye contact, shared smiles, and the pleasure of being together face-to-face. Concurrently, subjectivity develops within play as a space of personal and social creation where individuals express emotions, assume roles, and negotiate their place with others.

Research indicates that thematic social role-play expands the boundaries of the zone of proximal development (ZPD). This is achieved by co-constructing a joint play space that includes both adults and children (Bredikyte and Hakkarainen, 2017; Bredikyte and Brandisauskiene, 2023); in other words, role-play strengthens social and communicative interactions. Likewise, new forms of behavior emerge during play; for example, children learn to interact with others with empathy, respect, and compassion because they learn to imagine the other person's inner world (González-Moreno, 2023).

Therefore, an essential requirement for promoting child development at this stage is the adult's ability to scaffold thematic social role-play activities, guiding them from their simplest to their most complex forms. This type of play holds the greatest developmental potential for preschool-aged children, as it requires them to consider multiple perspectives to solve problems that arise during play.

For that reason, teachers must reflect on the theoretical concepts of the historical-cultural approach to analyze their practice and its impact on children's development. A critical examination of these concepts and their application in preschool education highlights the need to design theory-based thematic social role-play practices. These practices provide a deeper understanding of child development, ultimately transforming the teacher's role.

Implementing thematic social role-play requires time and collaborative teamwork within a supportive environment that involves parents. When educational institutions and families work together, children strengthen their social and emotional skills through mutual support. Furthermore, teachers require continuous theoretical, methodological and reflective training.

The theoretical proposal of the historical-cultural approach explains aspects of child development that traditional, purely biological, or “spontaneous” perspectives fail to clarify. Unlike frameworks that view play merely as entertainment, a release of energy, or an innate activity, this paradigm offers the following unique insights:

- Play as the driving force: structured play is both the cause and the engine of development. Through planned guidance, role-play fosters the psychological “neo-formations” essential for future school life.
- Mediated symbolic function: many schools of thought assume that the ability to use symbols (e.g., using a wooden ruler to represent a thermometer) emerges automatically with age. This framework challenges that view, showing that symbolic function is not spontaneous. Instead, it must be actively fostered by an adult through progressive developmental stages: the material level (using concrete substitutes), the perceptual level (using drawings or diagrams), and the verbal level (using only words). The historical-cultural approach rigorously explains how play facilitates the transition from object-based actions to abstract mental operations.
- Essential adult guidance: while traditional explanations argue that adults should not intervene in play to avoid “corrupting” creativity, the historical-cultural perspective holds that mature, complex play is not achieved in isolation. The adult acts as a guide who expands upon the child's activities, introduces play-based challenges, and actively broadens the Zone of Proximal Development (ZPD), transforming simple play into a powerful tool for school readiness.

The adult employs strategies for emotional connection to help the play progress to more complex levels. Some of these strategies include:

- To capture and collectively guide the children's attention toward the play activity, the adult uses facial expressions, body language, and strategic pauses. These pauses provide children with the necessary time to reflect before speaking or responding during the play.
- To help a child engage in the activity, the adult monitors and responds to their zone of proximal development through affectionate dialogue that fosters closeness.
- To maintain the child's engagement, the adult forms a play partnership with them, actively contributing to the co-regulation of emotions and social attitudes. Active adult involvement does more than just entertain; it transforms play into a powerful vehicle for psychological, social, and emotional development. The adult's presence acts as an “anchor” that extends the child's attention span during an activity. Through shared play, the adult can introduce manageable challenges that the child could not navigate independently, thereby fostering cognitive learning without inducing frustration. Feeling supported and emotionally attuned reduces the child's stress, enabling the brain to shift from a state of alertness to one of exploration and creativity.
- To stimulate creativity during play, the adult can use questions such as: “What can we do now?”, “What could we come up with next?”, “Where should our adventure go from here?”, “How can we change this right now?”, or “If the dragon is hungry, what can we cook to calm it down?” These types of questions break repetitive patterns and encourage the child to imagine entirely new scenarios, solutions, and perspectives.
- To enrich the play activity, the adult can prioritize a specific theme for deeper exploration. As play progresses, the children will naturally begin to suggest the themes that interest them most.

What children create together—as they transcend their immediate reality through play—belongs to them by right and shapes their identity. Cooperative play is not merely a reproduction of their environment but a space for transformation where children reinterpret the world. Therefore, understanding the nature of creative imagination and symbolic thought, alongside designing intentional strategies to foster them, constitutes two of the most significant and urgent pillars of contemporary child psychology and pedagogy.

These cognitive and affective dimensions should not be viewed as mere pastimes or transitional stages, but rather as genuine drivers of holistic development. Expanding this conceptual framework requires delving into three fundamental areas:

- Cultural appropriation and subjectivity: when children create together, they build a web of shared meanings. What originates on the interpsychological (social) level becomes part of their intrapsychological (individual) world; consolidating both their autonomy and their sense of group belonging.
- The symbol as a tool for thought: symbolic thought allows children to break free from the constraints of concrete objects (for example, transforming a wooden stick into a winged horse). This capacity for abstraction forms the bedrock for complex, higher psychological functions, including literacy, mathematical thinking, and logical-abstract reasoning.
- Imagination as the basis for future innovation: creative imagination in childhood recombines elements of past experiences to generate entirely new realities. Fostering this capacity at school and at home ensures the development of individuals who possess critical thinking, cognitive flexibility, and the ability to solve complex problems.

## Conclusions

The original theoretical contribution of thematic social role-play lies in how this activity allows for a deeper understanding of how creative imagination and symbolic thought take shape in preschool-aged children through communicative interactions. When analyzing the origins of these psychological formations within the context of play, it is essential to examine how they evolve into individual capabilities and stable components of the volitional structure of the child's personality.

Furthermore, recommendations for implementing social role-play aim to foster a deeper understanding of children's thoughts and ideas, thereby contributing to their development through interpersonal relationships. Through these shared activities, adults can re-evaluate their educational roles from a perspective closer to that of the child. This shift in focus helps design educational frameworks that align with the needs and capabilities of both the peer group and each individual child. Ultimately, role-play provides preschool children with an emotionally supportive environment that fosters the creation of strong, secure bonds.

The true mission of pedagogical intervention is to safeguard and enrich the space for symbolic play. By recognizing play as this primary setting, education equips children with the cognitive and socio-emotional resources to become active co-creators of their own futures.

To achieve this, educators must transform their pedagogical practices and integrate thematic social role-play into their daily routines. This shift requires the gradual development of skills needed to guide role adoption and to interact with children in creative and expansive ways. Consequently, designing and sustaining these activities presents a significant challenge. Nevertheless, overcoming this challenge is essential for fostering authentic, play-based learning environments.

Finally, although play focuses primarily on child development, it is worth noting that adults also experience professional and personal growth when engaging in play with children. This mutual dimension of development represents a promising avenue for future research.

To realize these benefits, educators must transform their pedagogical practices and integrate thematic social role-play into their daily routines. This shift requires the gradual development of the skills needed to guide role-taking and to interact with children in creative, expansive ways. Consequently, designing such activities remains a significant challenge. Nevertheless, overcoming this obstacle is essential for fostering complex child development and nurturing the psychological neoformations necessary for a successful transition to primary school.
